# Inter-Specific Coral Chimerism: Genetically Distinct Multicellular Structures Associated with Tissue Loss in *Montipora capitata*

**DOI:** 10.1371/journal.pone.0022869

**Published:** 2011-07-28

**Authors:** Thierry M. Work, Zac H. Forsman, Zoltán Szabó, Teresa D. Lewis, Greta S. Aeby, Robert J. Toonen

**Affiliations:** 1 Honolulu Field Station, National Wildlife Health Center, United States Geological Survey, Honolulu, Hawai'i, United States of America; 2 Hawai'i Institute of Marine Biology, Kāne'ohe, Hawai'i, United States of America; 3 Dexter Fish Health Unit, Dexter National Fish Hatchery and Technology Center, United States Fish and Wildlife Service, Dexter, New Mexico, United States of America; King Abdullah University of Science and Technology, Saudi Arabia

## Abstract

*Montipora* white syndrome (MWS) results in tissue-loss that is often lethal to *Montipora capitata*, a major reef building coral that is abundant and dominant in the Hawai'ian Archipelago. Within some MWS-affected colonies in Kane'ohe Bay, Oahu, Hawai'i, we saw unusual motile multicellular structures within gastrovascular canals (hereafter referred to as invasive gastrovascular multicellular structure-IGMS) that were associated with thinning and fragmentation of the basal body wall. IGMS were in significantly greater densities in coral fragments manifesting tissue-loss compared to paired normal fragments. Mesenterial filaments from these colonies yielded typical *M. capitata* mitochondrial haplotypes (CO1, CR), while IGMS from the same colony consistently yielded distinct haplotypes previously only found in a different *Montipora* species (*Montipora flabellata*). Protein profiles showed consistent differences between paired mesenterial filaments and IGMS from the same colonies as did seven microsatellite loci that also exhibited an excess of alleles per locus inconsistent with a single diploid organism. We hypothesize that IGMS are a parasitic cellular lineage resulting from the chimeric fusion between *M. capitata* and *M. flabellata* larvae followed by morphological reabsorption of *M. flabellata* and subsequent formation of cell-lineage parasites. We term this disease Montiporaiasis. Although intra-specific chimerism is common in colonial animals, this is the first suspected inter-specific example and the first associated with tissue loss.

## Introduction

Diseases inducing tissue loss have led to declines of dominant corals in Florida and the Caribbean [1], [2] and are generally thought to be caused by infectious agents. For example, convincing evidence exists experimentally and morphologically that black band in the Caribbean is caused by a consortium of bacteria and other organisms [3]. Several studies have implicated bacteria as causes of tissue loss in *Acropora* from the Caribbean [4], [5] and *Acropora* and *Pachyseris* the Pacific [6] based on the ability of these agents to replicate gross lesions of tissue loss experimentally. However, tissue loss is a non-specific gross lesion that can be associated with a wide variety of extrinsic organisms such as bacteria, ciliates, algae, fungi, crown of thorns starfish, snails, nudibranchs and flat worms [7], [8], [9], [10], [11]. This complicates determination of causation of lesions in corals based on gross examination alone [12], [13].

In contrast to extrinsic factors, much less is known about intrinsic factors associated with tissue-loss in corals. One example is the coral *Seriatopora hystrix* that undergoes a physiological process called polyp bail-out in response to predation or environmental stress resulting in rapid tissue loss over the entire colony [14]. Another possible example is “shut-down-reaction” in *Acropora* [15]. Intrinsic genetic factors associated with disease in corals have thus far not been documented. As part of an investigation of tissue-loss in a dominant coral, *Montipora capitata*, in Kane'ohe Bay, Oahu, Hawai'i, we documented pathology associated with a putative cellular parasite (referred to as montiporaiasis) that did not fit standard Cnidarian morphology [16]. We present here molecular and protein data that reveal unexpected differences between these putative parasites and mesenterial tissues from the same colony. Based on the combined data, we hypothesize IGMS to be somatic or germ cell lineage parasites formed by inter-species chimerism between two reef corals, M *capitata* and *Montipora flabellata*.

## Results

**Figure 1:**
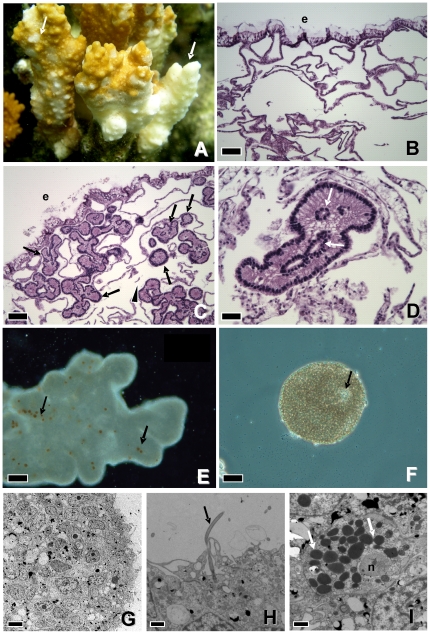
*Montipora capitata* gross lesion (A), hematoxylin and eosin (B–D), darkfield (E), phase contrast (F), and electron micrographs (G–I). A) coral manifesting gross lesions associated with IGMS; note multiple variably sized distinct amorphous area of multifocal to diffuse tissue loss revealing bare white skeleton (white arrows). B) Normal coral. Note mesenterial filaments; bar = 100 µm. C) Coral with IGMS. Note numerous round to pleomorphic IGMS (arrows) within gastrovascular canals effacing basal body wall that manifests atrophy and fragmentation (arrowhead); bar = 100 µm. D) Higher magnification of IGMS. Note cavities lined by cuboidal cells (arrow); bar = 20 µm. E) Darkfield micrograph of amorphous IGMS. Note brown pigment granules (arrow); bar = 50 µm. F) Phase contrast micrograph of round IGMS. Note what may be internal cavity (arrow); bar = 50 µm. G) IGMS. Note aggregation of cells with no apparent organization, lack of gonads, nematocysts, gut, or mesoglea. Bar = 10 µm. H) Rare cilia with basal body (arrow) on surface of IGMS; bar = 1 µm. I) Presumed pigment cell in IGMS. Note variably-sized elliptical to round intracytoplasmic electron-dense granules (arrow); bar = 1 µm. n = Nucleus, e = epidermis.

Coral fragments with high densities of IGMS manifested focal to multifocal indistinct acute tissue-loss revealing bare white skeleton (Fig. 1A). On microscopy, IGMS were located within gastrovascular canals and when present in high numbers resulted in fragmentation or effacement of basal body walls; however, cell necrosis was not present (Fig. 1B–C). IGMS were round to amorphous, contained occasional pigment cells, and ranged from 85–350 µm at their widest point. Occasionally, central cavities lined by cuboidal cells were seen (Fig. 1D–F). Masson's trichrome failed to reveal collagen within the IGMS, and no zooxanthellae, cnidae (nematocysts), or gonads were seen on light microscopy. On electron microscopy, IGMS consisted of amorphous masses of cells with no mesoglea, gut, or other evident organized internal structures (Fig. 1G); occasional cilia with characteristic basal body were observed (Fig. 1H) as were what appeared to be pigment cells (Fig. 1I). Characteristics of mesenterial filaments and IGMS are summarized in Table 1.

**Table 1:** Morphologic characteristics of mesenterial filaments versus invasive gastrovascular multicellular structures (IGMS) in *M. capitata*.

Character	Mesenterial filament	IGMS
Zooxanthellae	Present	Absent
Cilia	Present	Rare
Pigment granules	Absent	Present
Gonads	Present	Absent
Mesoglea	Present	Absent
Myonemes	Present	Absent
Eosinophilic granular cells	Present	Absent
Lumen	Absent	Occasional
Location	Base of polyps	Throughout gastrovascular canals
Shape	Coiled or tortuous	Round to amorphous

**Figure 2:**
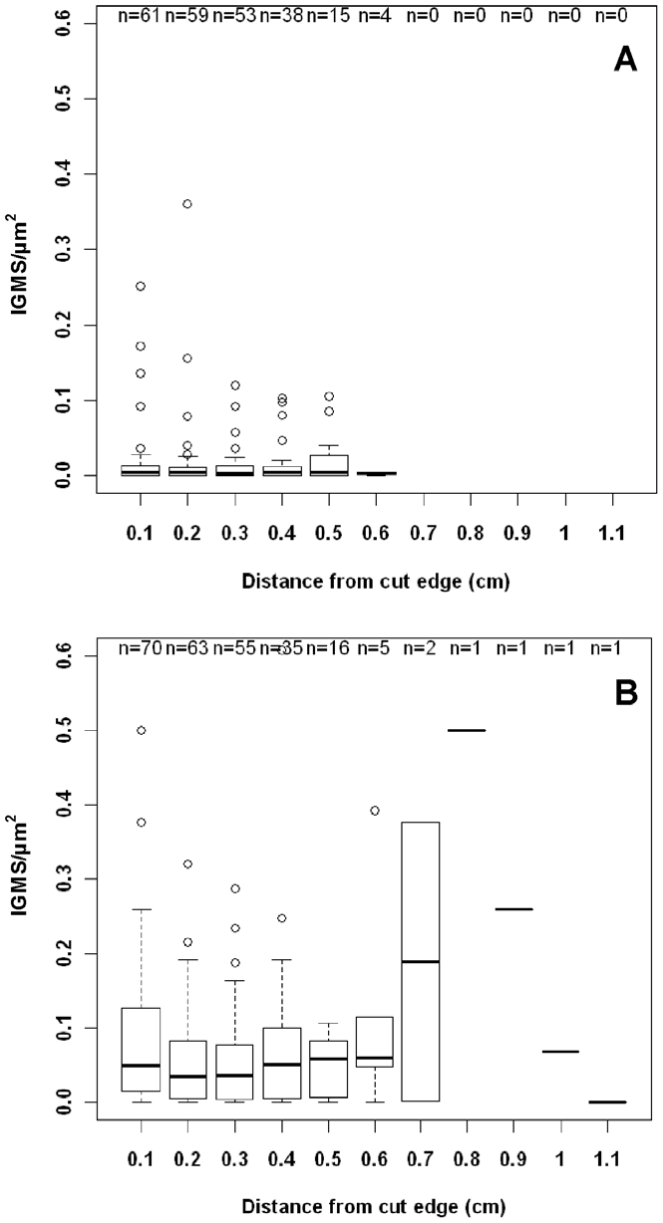
Box-whisker plots of invasive gastrovascular multicellular structures (IGMS)/µm2 with distance from broken fragment edge of normal tissues (A) or distance from edge of lesion in tissues manifesting WS (B). Sample size indicated above each box. Bold line indicates median, lower and upper margin of box are first and third quartile, lower and upper whiskers indicate lowest and highest data point within 1.5*interquartile range, respectively, and dots indicate outliers.

Of 46 colonies with *Montipora* white syndrome (MWS) examined at multiple time points over one year, prevalence of IGMS ranged from 0 to 34%. Significantly higher densities of IGMS were present in tissues with lesions (0.07±0.08 IGMS/µm2) compared to normal tissues from the same colony (0.01±0.03 IGMS/µm2) (Mann-Whitney U = 214, p = 0.004), and IGMS were present in consistently higher densities and deeper within tissues (Fig. 2).

Attempts to infect colonies with IGMS either through direct contact or water in aquaria failed, and experimental fragments with IGMS invariably lost all their tissues within 6–8 weeks. However; in two separate experimental open water table studies to monitor healing of experimentally induced lesions in lesion-free *M. capitata*, low densities (1–3 IGMS/tissue section) were observed on histology (100% of 80 fragments in the first experiment and 10% of 80 fragments in the second experiment); all fragments subsequently healed completely.

**Figure 3:**
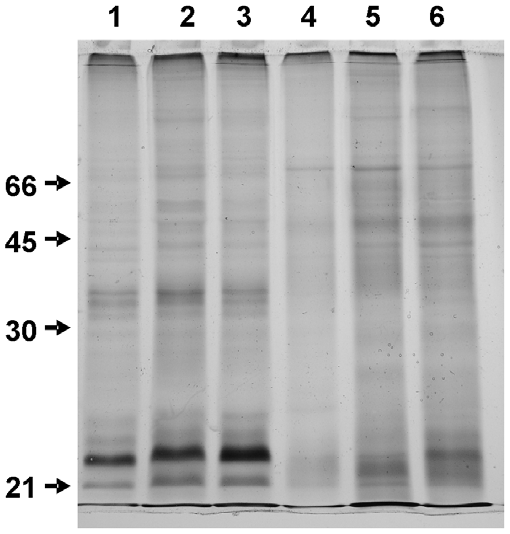
Denaturing polyacrylamide gel electrophoresis of invasive grastrovascular multicellular structures (IGMS) (lanes 1–3) for paired mesenterial filaments (Lanes 4–6) from coral fragments from three different infected colonies. Note protein bands at ca. 25 and 35 KD for IGMS not present in mesenterial filaments.

**Figure 4:**
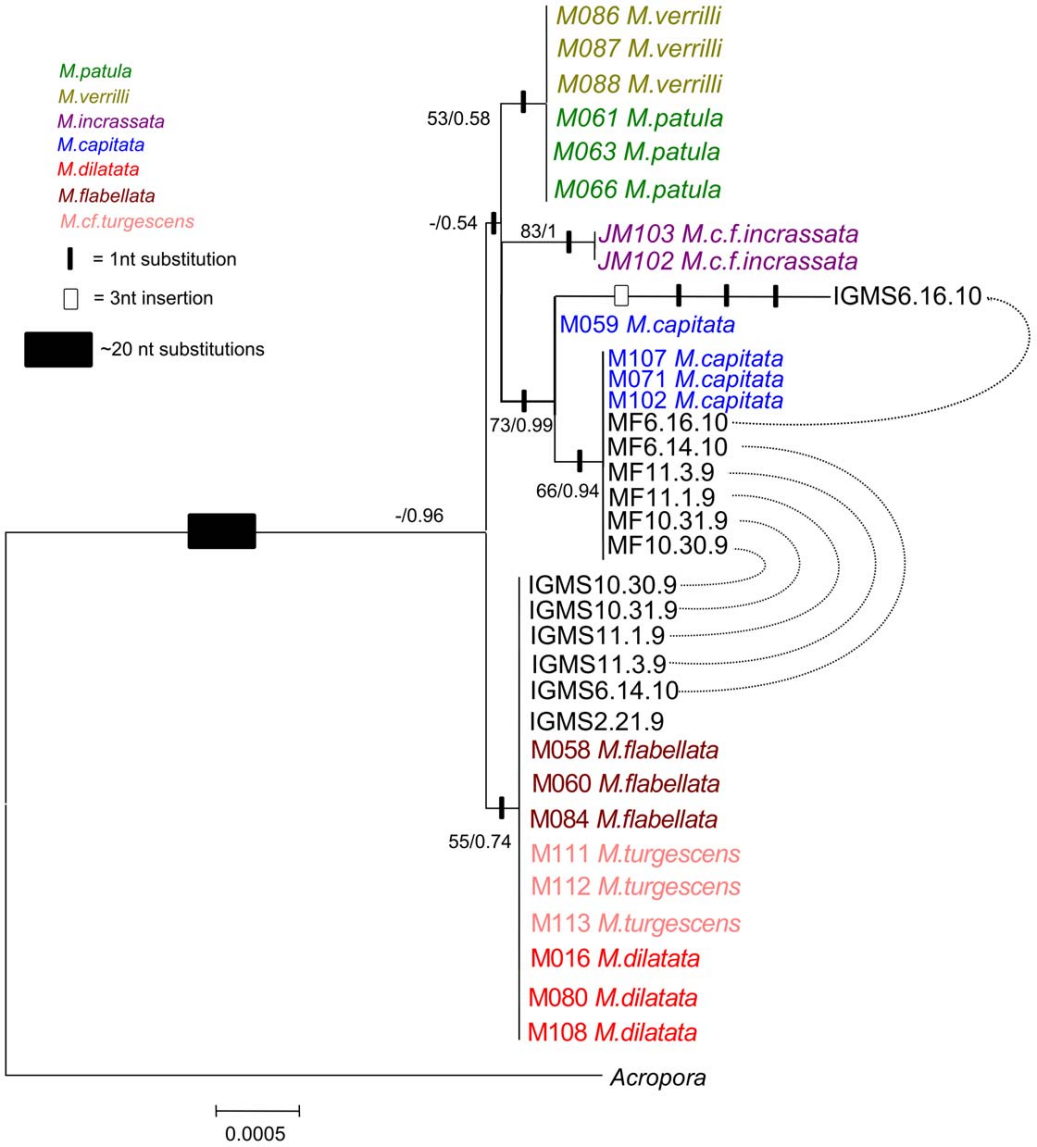
Bayesian inference (BI) tree of the mitochondrial CO1 sequences for Hawaiian specimens. Maximum likelihood and BI confidence values are shown. Connecting lines indicate mesenterial filaments and putative invasive gastrovascular multicellular structures (IGMS) from the same colony indicating distinct genetic types.

**Figure 5:**
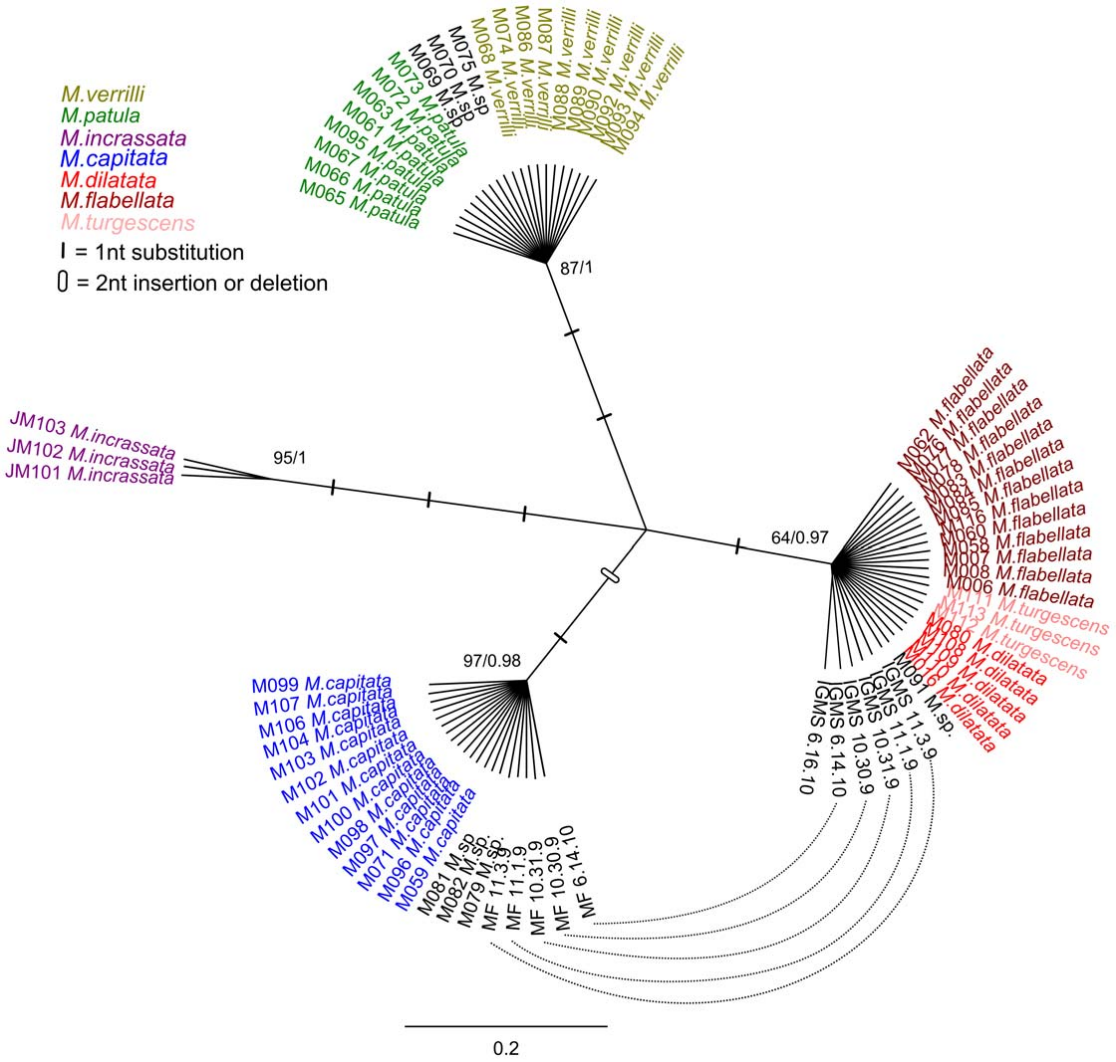
Bayesian inference (BI) tree of the mitochondrial control region for Hawaiian specimens, color-coded by species. Maximum likelihood and BI confidence values are shown. Solid rectangles indicate a single nucleotide substitution, open rectangles indicate a two nucleotide insertion or deletion. Dashed lines indicate mesenterial filaments and invasive gastrovascular multicellular structures (IGMS) tissue types that originated from the same colony.

When coral fragments were incubated overnight with salt agar, IGMS migrated 0.1–4 cm from within the fragments into surrounding sterile seawater. Compared to mesenterial filaments, IGMS had a different protein profile; bands unique to IGMS were seen between 20–45 KD whereas bands unique to mesenterial filaments were seen at >45KD (Fig. 3). The initial survey (DNA extraction from IGMS on 4/21/2009) with ‘universal’ primers yielded sequences for 18S [17], CO1 [18], and 16S [19], with strong similarity to close relatives of *Montipora* (93% to *Montipora peltiformis* AY722777.1, and 100% to *Anacropora matthai* AY903295.1, respectively) according to the National Center Biological Information (NCBI) basic local alignment search tool [20], [21]. We observed that the CO1 sequence from IGMS tissue unexpectedly differed from Hawaiian *M. capitata* from a previous study [22] and was 100% identical to haplotypes that have previously only been isolated from *M. flabellata* or from the very rare species *M. dilatata*, or *M. cf. turgescens* (Fig. 4). The second independent experiment extracted DNA from freshly isolated IGMS (1/11/2010) and confirmed this result; all mesenterial filaments shared identical CO1 haplotypes with other *M. capitata* samples, while all IGMS haplotypes from the same colonies were identical to *M. flabellata*. The same pattern was observed for the mitochondrial control region for all four colonies (NCBI #HQ246454-HQ246712) (Fig. 5).

Microsatellite results further confirmed that mesenterial filaments and IGMS tissue types from each colony were genetically distinct. Most loci differed by several alleles between tissue types; on average there were fewer shared microsatellite alleles than different ones (overall average bands shared = 1.7±0.5 SD, overall average bands different = 1.9±0.3 SD); (Table S1, S2). All loci except Mc0947 showed multiple alleles per locus (overall average = 3.8±0.3 SD) per colony, a pattern inconsistent with a single diploid organism. Each tissue type also contained an excess of alleles per locus; mesenterial filaments average = 2.2±0.7 SD; IGMS average = 3.3±0.4 SD that has not been previously observed for other *M. capitata* samples using these markers [23]. This pattern is consistent either with polyploidy or a mixed sample containing several individuals. These microsatellite results were highly consistent over repeat runs and multiple independent isolations and extractions of IGMS and mesenterial filaments from affected colonies, whereas no peaks were observed in any of the negative controls.

## Discussion

Within affected *M. capitata* colonies, we saw unusual multicellular structures (IGMS) that contained mitochondrial CO1 and control region sequences that differed from that of mesenterial filaments from the same colony in terms of morphologic, protein, and genetic profiles. IGMS were not transmissible experimentally, wound repair experiments in healthy *M. capitata* failed to reveal IGMS as a simple host response to trauma, and IGMS were expelled or capable of movement out of semi-submerged coral fragments placed on salt agar such that they could be collected in isolation from the host coral tissue. Finally, IGMS appeared to be harmful to the coral host as they were consistently associated with thinning and fragmentation of the basal body wall. The IGMS mitochondrial haplotypes differed from *M. capitata* (CO1 n = 6, CR n = 6) and were identical to the reef coral, *M. flabellata* (or to the very rare *M. dilatata* or *M.cf. turgescens*) with the exception of a single CO1 sequence (IGMS6.16.10) that was also divergent from other *M. capitata* haplotypes (Figure 5). Microsatellite markers and protein profiles indicated consistent differences across most loci between paired mesenterial filaments and IGMS samples from the same colony, and the microsatellite profiles for both tissue types were inconsistent with a single diploid organism. This result was surprising given that no such issue was detected in a sample of 560 colonies genotyped from 13 locations across the length of the Hawai'ian Archipelago [23].

Given these combined observations, we propose that IGMS are cell-lineage parasites that are the result of chimeric fusion between *M. capitata* and *M. flabellata* larvae followed by morphological re-sorption of *M. flabellata*. Chimerism (the fusion of two or more post-zygotic individuals) has been documented in nine phyla; most commonly in colonial marine animals such as corals, bryozoans, and ascidians [24], [25]. Newly settled colonial animals typically have high mortality and gregarious settling, and larval chimerism is thought to be a mechanism to rapidly increase size, survivorship, and genetic variation in a heterogeneous environment [26], [27], [28], [29], [30], [31]. Somatic or germ lineage parasitism is a potential negative consequence of chimerism, whereby a dominant genotype reabsorbs the tissue of a subordinate that can then persist in the form of totipotent stem cells that can later invade and parasitize either the somatic or germ tissue [25], [32], [33], [34], [35].

Allorecognition systems are thought to have emerged as a defense against cell lineage parasitism; however, in colonial animals this mechanism is less active and potentially error prone during early stages of development [36], [37]. For example in the coral *Stylophora pistillata*, non-related larvae fused and formed stable chimeras, while colonies older than 2 months were incompatible [38]. Intra-specific chimerism is common in soft coral [39] and has been well documented in stony corals, most notably in *S. pistillata* [38], [40], *Pocillopora damicornis* [29], and *Acropora millepora* [41], a sister genus to *Montipora*. Up to 47% of *A. millepora* larvae fused under lab conditions and microsatellite markers revealed that as many as 5% of the colonies in nature were composed of two or more closely related genotypes [41].

Inter-specific chimerism has been experimentally induced in a variety of organisms including vertebrates [42] and plants [43]. This is the first instance to our knowledge of suspected inter-specific animal chimerism; however; coral species boundaries are difficult to delineate and in some cases may be semi-permeable due to hybridization. Confirming this hypothesis will require more targeted studies, but if confirmed, corals affected by this disorder are likely to serve as a useful model.

If the chimera hypothesis is true, it is not yet clear what triggers the onset of tissue loss in *M. capitata*. Temperature may be a factor. For example, clonal urochordates (*Botryllus schlosseri*) have shifts in chimeric constituents in accordance with changes in seawater temperature [40]. The exact role that IGMS play in tissue loss in *M. capitata* is unclear, but IGMS were more abundant in coral fragments manifesting tissue-loss. Parasites do not always kill their hosts, and animals that appear healthy can harbor parasitic infections [44] which would be consistent with the presence of IGMS in both healthy and compromised coral. The pathogenesis of montiporaiasis is thus fertile ground for further study.

There are alternative hypotheses for IGMS; however, they do not reconcile with all of the data. Hybridization between *M. capitata* and *M. flabellata* (or the rare *M. dilatata* or *M. cf. turgescens*) could potentially result in multiple tissue types with differing mitochondrial genotypes. This is similar to mitochondrial heteroplasmy and doubly uniparental inheritance (DUI) observed in mussels (*Mytilus* sp.), where male gonads contain a distinct mitochondrial genotype [45], [46]. The microsatellite results (SI text), however, are inconsistent with hybridization because most loci differ between mesenterial filaments and IGMS within individual colonies. Furthermore, the regions where *M. capitata* were sampled were in South Kane'ohe Bay whereas *M. flabellata* are concentrated in the north bay, only a handful of *M. dilatata* colonies exist in that area, and *M. cf. turgescens* has only been documented in the Northwest Hawai'ian Islands [47]. We have not thus far documented *M. flabellata* in the vicinity of affected colonies, but montiporaiasis affects ca. 20% of *M. capitata* colonies manifesting tissue loss within Kane'ohe Bay. We have no data on the overall prevalence of IGMS in *M. capitata* that do not manifest tissue loss.

Allopolyploidy may result in more than two alleles per locus per individual; however, the genotype should remain consistent across tissues, and the maximum number of alleles should be relatively consistent among individuals. The maximum number of alleles per locus is highly variable among tissue types with a colony and between individuals which is more consistent with a mixture of several diploid individuals. Furthermore, a population genetic survey of 560 colonies sampled across the length of the Hawai'ian Archipelago found that virtually all had only 2 alleles per locus, and all but 2 loci with null alleles conformed to Hardy-Weinberg expectations [23]. Contamination, mislabeling or other procedural artifacts are highly unlikely because the results were highly concordant across mitochondrial, microsatellite and protein assays in spite of the fact that multiple collections, purifications, DNA extractions and amplifications were done by separate workers. For the microsatellite assay, all samples were run twice and were highly consistent, and there were no signs of amplification in the negative controls done on the ABI 3100 Genetic Analyzer which is highly sensitive to minute amounts of DNA. Known PCR artifacts such as nonspecific amplification primer binding, or slip-strand mispairing are inconsistent with the genetic results presented here.

More broadly, these findings may be the first example of chimerism between two coral species and the first implication of chimerism resulting in coral disease. In addition to elucidating their role in causing tissue-loss in *M. capitata*, this phenomenon may be a valuable system for research on the evolution of multicellularity and allorecognition systems in corals in general. Finally, the presence of multiple genotypes from different species within a single coral colony has the potential to confound a wide variety of studies, particularly phylogenetic, population genetic, and proteomic studies. It is also likely to add to the difficulty in distinguishing between coral species and in understanding the role of hybridization in the evolution of reef-building corals. Accordingly, it would be wise for such studies to incorporate microscopic examination of tissues to at least rule out the presence of cellular lineage parasites that could affect genomic investigations.

## Materials and Methods

Paired fragments (normal and lesion tissue) were collected from *M. capitata* colonies manifesting tissue loss [12] and fixed in zinc formalin (Z-Fix, Anatech) diluted in seawater according to manufacturer instructions. Coral fragments were subsequently decalcified (Cal Ex II, Fisher Scientific), embedded in paraffin, trimmed at 5 µm, and stained with hematoxylin and eosin. As appropriate, Massons trichrome stain was used to highlight collagen in the sections. For each of 46 colonies, sampled over a one-year period, IGMS were quantified in paired tissue sections with and without lesions by counting numbers of IGMS/µm^2^ along a gradient from the lesion inwards in 100 µm intervals. Mean IGMS densities were compared between normal and lesion fragments using the Mann-Whitney U test because the data did not meet assumptions of normality and equal variance.

For electron microscopy, IGMS were collected from live coral by partially immersing a coral fragment in artificial seawater in a petri dish, placing a block of agar made with saturated NaCl on top of the fragment, and incubating at room temperature overnight. This provided a high salt/low moisture gradient prompting the IGMS to migrate or be expelled from the fragment to surrounding artificial seawater from which they were harvested the next morning (salt method). IGMS were fixed in Trump's fixative [48], post fixed in 2% osmium tetroxide, embedded in epoxy, and cut into 1 µm thick toluidine blue-stained sections. Ultrathin sections were stained first with uranyl acetate then lead citrate and examined using a Zeiss LEO 912 transmission electron microscope at the University of Hawai'i Core Electron Microscopy Facility. Fresh IGMS collected by salt method were measured under light microscopy (100×) along their longest axis using an ocular micrometer calibrated to a stage micrometer.

To assess whether IGMS were transmissible, a fragment from IGMS-infected colonies was incubated with two IGMS-free fragments from a healthy colony (infection/non-infection was confirmed by histology) either in direct contact or in water contact in closed 11 L aquaria filled with artificial seawater maintained at 27°C on a 12∶12 LD cycle; a duplicate tank with 3 healthy histologically-confirmed uninfected fragments served as a control. Experiments were ended if tissue-loss developed in healthy contact or non-contact fragments or when the IGMS-laden fragment died completely. At termination of the experiments, all tissues were examined by histology for the presence of IGMS. Transmission experiments were replicated three times with separate colonies (27 fragments total examined). To determine if IGMS were a host response to trauma, 80 fragments from a healthy colony were experimentally traumatized by abrading a 1 cm^2^ area of tissue and skeleton and monitoring the wound repair process using histology every 2–4 days for 32 days.

For protein and molecular studies, paired IGMS and extruded mesenterial filaments were collected from a single affected coral fragment from each of three different colonies using the salt method, harvested, washed extensively with sterile artificial seawater, pelleted by centrifugation, and seawater decanted prior to freezing (−70°C). For proteins, IGMS and mesenterial filaments were homogenized separately in 10 volumes of phosphate buffered saline, centrifuged, the supernates resolved on 12% denaturing polyacrylamide, and bands visualized with silver [49]. DNA was extracted on three separate occasions, by three separate workers on IGMS and mesenterial filaments isolated from a single fragment as previously described. In total there were 6 different colonies (2 colonies/worker) with paired IGMS and mesenterial filaments extracted using the Qiagen DNeasy Blood and Tissue kit (Tissue protocol) following the manufacturers recommendations. PCR primers were based on previously published primers: mt16S, 1 TCGACTGTTTAGMAAAAACATA, 2 ACGGAATGAACTCAAATCATGTAAG [19], mtCO1, HCO2198 TAAACTTCAGGGTGAGMAAAAAATC, LCO1490 GGTCAACAAATCATAAAGATATTGG, [18]; mtCR, Ms FP2 TAGACAGGGGMAAGGAGAAG, MON RP2 GATAGGGGCTTTTCATTTGTTTG [17].

PCR reactions were performed on a MyCycler thermal cycler (BioRad). Each PCR contained 1 µL of DNA template, 2.5 µL of 10× ImmoBuffer, 0.1 µL IMMOLASE DNA polymerase (Bioline), 3 mm MgCl2, 10 mm total dNTPs, 13 pmol of each primer, and molecular biology grade water to 25 µL final volume. Hot-start PCR amplification conditions varied slightly depending on the primer set used and were generally: 95°C for 10 min (1 cycle), 95°C for 30 s, annealing temperature (2 degrees less than primer melting temperature, ranging between 50 and 60°C) for 30 s, and 72°C for 60 s (35 cycles) followed by a final extension at 72°C for 10 min (1 cycle). PCR products were visualized using 1.0% agarose gels (1× TAE) stained with Gelstar®. PCR products for direct sequencing were treated with 2 U of exonuclease I and 2 U of shrimp alkaline phosphatase (Exo∶SAP) using the following thermocycler profile: 37°C for 60 min, 80°C for 10 min. Treated PCR products were then cycle-sequenced using BigDye Terminators (Perkin Elmer) run on an ABI 3130XL automated sequencer at the NSF-EPSCoR core genetics facility at the Hawai'i Institute of Marine Biology (HIMB). Resulting sequences were inspected and aligned using Geneious Pro 4.8.5 [50] to implement either ClustalW [51] or Muscle [52]. The nucleotide substitution model was selected in Modeltest V.3.7 [53] by the Akaike information criterion (COI = K81uf+I). All phylogenetic analyses were performed with Bayesian Inference (BI) and Maximum Likelihood (ML). Bayesian Inference trees were generated with Mr.Bayes 3.1.2 [54], with 1,100,000 generations and a burn in of 110,000 generations, and ML trees were generated by RaxML [55].

Microsatellite markers, PCR conditions, primer tailing method, and pooling methods were done as described previously [56]. The amplified fragments were analyzed on the ABI 3100XL Genetic Analyzer at the EPSCoR core genetics facility at HIMB and sized using GENEMAPPER v4.0 and GS500LZ size standards (Applied Biosystems). Bands were scored as present only in cases where a clearly visible peak was determined to be at least ½ the height of the size standard. Reactions that failed to produce any clear peaks were scored as ‘na’ (Appendix 1).

## Supporting Information

Table S1 **Table of sample collection and sequencing information. Check marks indicate successfully sequenced samples.** Abbreviations: G.C. = Greg Concepcion, R.H. = Roxanne Haverkort, Z.F. = Zac Forsman, E.C. = Evelyn Cox, C.H. = Cynthia Hunter, M.T. = Molly Timmers, I.B. = Iliana Baums, T.W. = Thierry Work, J.M. = Jim Maragos.
(DOC)

Table S2 **Microsatellite presence or absence score sheet.** Size in base pairs of bands at each locus (Mc0004-Mc0947). Absence of bands are indicated by an X, f indicates a failed reaction. Each colony is represented by a number indicating month and day of SCP isolation. Each sample was run twice (a,b). Gray columns indicate confirmed differences between MF and SCP for a given colony.
(XLS)
