# Supplementary material for: Inter-Specific Coral Chimerism: Genetically Distinct Multicellular Structures Associated with Tissue Loss in Montipora capitata
Source: PLoS One. 2011 Jul 28;6(7):e22869. doi: 10.1371/journal.pone.0022869 (PMC3145771; doi:10.1371/journal.pone.0022869)
Supplement: Table S1 — Table of sample collection and sequencing information. Check marks indicate successfully sequenced samples. Abbreviations: G.C. = Greg Concepcion, R.H. = Roxanne Haverkort, Z.F. = Zac Forsman, E.C. = Evelyn Cox, C.H. = Cynthia Hunter, M.T. = Molly Timmers, I.B. = Iliana Baums, T.W. = Thierry Work, J.M. = Jim Maragos. (DOC) [file pone.0022869.s001.doc]

| **Code** | **Species** | **Location** | **Collector** | **COX1** | **CR** |
| --- | --- | --- | --- | --- | --- |
| M016 | *M. dilatata* | Kaneohe Bay, Oahu | C.H. | ✓ | ✓ |
| M058 | *M. flabellata* | Magic Island, Oahu | G.C.;R.H. | ✓ | ✓ |
| M059 | *M. capitata* | Magic Island, Oahu | G.C.;R.H. | ✓ | ✓ |
| M060 | *M. flabellata* | Magic Island, Oahu | G.C.;R.H. | ✓ | ✓ |
| M061 | *M. patula* | Magic Island, Oahu | G.C.;R.H. | ✓ | ✓ |
| M062 | *M. flabellata* | Magic Island, Oahu | G.C.;R.H. |  | ✓ |
| M063 | *M. patula* | Magic Island, Oahu | G.C.;R.H. | ✓ | ✓ |
| M065 | *M. patula* | Lanikai, Oahu | G.C.;R.H. |  | ✓ |
| M066 | *M. patula* | Lanikai, Oahu | G.C.;R.H. | ✓ | ✓ |
| M067 | *M. patula* | Lanikai, Oahu | G.C.;R.H. |  | ✓ |
| M068 | *M. verrilli* | Lanikai, Oahu | G.C.;R.H. |  | ✓ |
| M069 | *M.* sp*.* | Lanikai, Oahu | G.C.;R.H. |  | ✓ |
| M070 | *M.* sp. | Lanikai, Oahu | G.C.;R.H. |  | ✓ |
| M071 | *M. capitata* | Lanikai, Oahu | G.C.;R.H. | ✓ | ✓ |
| M072 | *M. patula* | Lanikai, Oahu | G.C.;R.H. |  | ✓ |
| M073 | *M. patula* | Lanikai, Oahu | G.C.;R.H. |  | ✓ |
| M074 | *M. verrilli* | Lanikai, Oahu | G.C.;R.H. |  | ✓ |
| M075 | *M.* sp. | Lanikai, Oahu | G.C.;R.H. |  | ✓ |
| M076 | *M. flabellata* | Kaneohe Bay, Oahu | G.C.;R.H. |  | ✓ |
| M077 | *M. flabellata* | Kaneohe Bay, Oahu | G.C.;R.H. |  | ✓ |
| M078 | *M. flabellata* | Kaneohe Bay, Oahu | G.C.;R.H. |  | ✓ |
| M079 | *M*. sp. | Kaneohe Bay, Oahu | J.S. |  | ✓ |
| M080 | *M. dilatata* | Kaneohe Bay, Oahu | C.H.;Z.F. | ✓ | ✓ |
| M081 | *M.* sp. | Pukoo, Molokai | C.H.;Z.F. |  | ✓ |
| M082 | *M.* sp. | Pukoo, Molokai | C.H.;Z.F. |  | ✓ |
| M083 | *M. flabellata* | Kaneohe Bay, Oahu | E.C. |  | ✓ |
| M084 | *M. flabellata* | Kaneohe Bay, Oahu | E.C. | ✓ | ✓ |
| M085 | *M. flabellata* | Kaneohe Bay, Oahu | E.C. |  | ✓ |
| M086 | *M. verrilli* | Oahu, Kaneohe Bay | C.H.;Z.F.;R.H. | ✓ | ✓ |
| M087 | *M. verrilli* | Oahu, Kaneohe Bay | C.H.;Z.F.;R.H. | ✓ | ✓ |
| M088 | *M. verrilli* | Oahu, Kaneohe Bay | C.H.;Z.F.;R.H. | ✓ | ✓ |
| M089 | *M. verrilli* | Oahu, Kaneohe Bay | C.H.;Z.F.;R.H. |  | ✓ |
| M090 | *M. verrilli* | Oahu, Kaneohe Bay | C.H.;Z.F.;R.H. |  | ✓ |
| M091 | *M.* sp. | Oahu, Kaneohe Bay | C.H.;Z.F.;R.H. |  | ✓ |
| M092 | *M. verrilli* | Oahu, Kaneohe Bay | C.H.;Z.F.;R.H. |  | ✓ |
| M093 | *M. verrilli* | Oahu, Kaneohe Bay | C.H.;Z.F.;R.H. |  | ✓ |
| M094 | *M. verrilli* | Oahu, Kaneohe Bay | C.H.;Z.F.;R.H. |  | ✓ |
| M095 | *M. patula* | Oahu, Kaneohe Bay | C.H.;Z.F.;R.H. | ✓ | ✓ |
| M096 | *M. capitata* | Maro Reef, NWHI | I.B. |  | ✓ |
| M097 | *M. capitata* | Mare Reef, NWHI | I.B. |  | ✓ |
| M098 | *M. capitata* | Maro Reef, NWHI | I.B. |  | ✓ |
| M099 | *M. capitata* | Pearl and Hermes, NWHI | G.C. |  | ✓ |
| M100 | *M. capitata* | Kure Atoll, NWHI | G.C. |  | ✓ |
| M101 | *M. capitata* | Lisianski, NWHI | G.C. |  | ✓ |
| M102 | *M. capitata* | Lisianski, NWHI | G.C. | ✓ | ✓ |
| M103 | *M. capitata* | Lisianski, NWHI | G.C. |  | ✓ |
| M104 | *M. capitata* | Pearl and Hermes, NWHI | G.C. |  | ✓ |
| M106 | *M. capitata* | Pearl and Hermes, NWHI | G.C. |  | ✓ |
| M107 | *M. capitata* | Kure Atoll, NWHI | G.C. | ✓ | ✓ |
| M108 | *M. dilatata* | Waikiki Aquarium, Oahu | G.C. |  | ✓ |
| M109 | *M. dilatata* | Waikiki Aquarium, Oahu | G.C. |  | ✓ |
| M110 | *M. dilatata* | Waikiki Aquarium | G.C. |  | ✓ |
| M111 | *M. cf. turgescens* | Pearl and Hermes, NWHI | M.T. | ✓ | ✓ |
| M112 | *M.cf. turgescens* | Lisianski, NWHI | M.T. | ✓ | ✓ |
| M113 | *M. cf. turgescens* | Pearl and Hermes, NWHI | M.T. | ✓ | ✓ |
| JM101 | *M. cf. incrassata* | Kawai, Hawaii | J.M. |  | ✓ |
| JM102 | *M. cf. incrassata* | Kawai, Hawaii | J.M. | ✓ | ✓ |
| JM103 | *M. cf. incrassata* | Kawai, Hawaii | J.M. | ✓ | ✓ |
| 2.21.09 | *M. capitata* | Kaneohe Bay, Oahu | T.W. | ✓ |  |
| 10.30.09 | *M. capitata* | Kaneohe Bay, Oahu | T.W. | ✓ | ✓ |
| 10.31.09 | *M. capitata* | Kaneohe Bay, Oahu | T.W. | ✓ | ✓ |
| 11.01.09 | *M. capitata* | Kaneohe Bay, Oahu | T.W. | ✓ | ✓ |
| 11.02.09 | *M. capitata* | Kaneohe Bay, Oahu | T.W. | ✓ | ✓ |
| 6.14.10 | *M. capitata* | Kaneohe Bay, Oahu | T.W. | ✓ | ✓ |
| 6.16.10 | *M. capitata* | Kaneohe Bay, Oahu | T.W. | ✓ |  |
